# RNA-sequencing analysis of shell gland shows differences in gene expression profile at two time-points of eggshell formation in laying chickens

**DOI:** 10.1186/s12864-019-5460-4

**Published:** 2019-01-25

**Authors:** Samiullah Khan, Shu-Biao Wu, Juliet Roberts

**Affiliations:** 1 Animal Science, School of Environmental and Rural Science, University of New England, Armidale, New South Wales 2351 Australia; 2 Present address: School of Animal and Veterinary Sciences, The University of Adelaide, Roseworthy, South Australia 5371 Australia

**Keywords:** Chicken oviduct, Eggshell formation, Ions transport, Gene regulation, Transcriptome profiling, Matrix proteins

## Abstract

**Background:**

Eggshell formation takes place in the shell gland of the oviduct of laying hens. The eggshell is rich in calcium and various glycoproteins synthesised in the shell gland. Although studies have identified genes involved in eggshell formation, little is known about the regulation of genes in the shell gland particularly in a temporal manner. The current study investigated the global gene expression profile of the shell gland of laying hens at different time-points of eggshell formation using RNA-Sequencing (RNA-Seq) analysis.

**Results:**

Gene expression profiles of the shell gland tissue at 5 and 15 h time-points were clearly distinct from each other. Out of the 14,334 genes assessed for differential expression in the shell gland tissue, 278 genes were significantly down-regulated (log_2_ fold change > 1.5; FDR < 0.05) and 413 genes were significantly up-regulated at 15 h relative to the 5 h time-point of eggshell formation. The down-regulated genes annotated to Gene Ontology (GO) terms showed anion transport, synaptic vesicle localisation, organic anion transport, secretion and signal release as the five most enriched terms. The up-regulated gene annotation showed regulation of phospholipase activities, alanine transport, transmembrane receptor protein tyrosine kinase signalling pathway, regulation of blood vessels diameter and 3, 5-cyclic nucleotide phosphodiesterase activity as the five most enriched GO terms. The putative functions of genes identified ranged from calcium binding to receptor activity. Validation of RNA-Seq results through qPCR showed a positive correlation.

**Conclusions:**

The down-regulated genes at 15 h relative to the 5 h time-point were most likely involved in the transport of molecules and synthesis activities, initiating the formation of the eggshell. The up-regulated genes were most likely involved in calcium transportation, as well as synthesis and secretory activities of ions and molecules, reflecting the peak stage of eggshell formation. The findings in the current study improve our understanding of eggshell formation at the molecular level and provide a foundation for further studies of mRNA and possibly microRNA regulation involved in eggshell formation in the shell gland of laying hens.

**Electronic supplementary material:**

The online version of this article (10.1186/s12864-019-5460-4) contains supplementary material, which is available to authorized users.

## Background

In laying hens, eggshell formation takes place in the shell gland region of the oviduct over an 18 h period [1]. The eggshell is composed of six distinct layers having calcite as a main component [2]. The calcium and bicarbonate ions contributing to the calcite (calcium carbonate) are secreted from the epithelial cells of the mucosa of the shell gland (reviewed in Hincke et al.) [3]. In addition to other regulators and transporters, the calbindin (*CALB1*) gene is involved in Ca^2+^ transportation across the cell membrane for eggshell formation [4]. Calcification within the shell gland is associated with stimuli initiated by ovulation or by neuroendocrine factors that control and coordinate both ovulation and calcium secretion [5]. Calcification of the egg first occurs slowly, increases to a rate of up to 300 mg/hr. over a duration of 15 h, and then again slows during the last 2 h before oviposition [5]. A higher rate of eggshell calcification may be correlated with a significantly higher level of calbindin mRNA expression that peaks at 16 h compared with 0–4.5 h of post-oviposition time [6]. While the expression level of the calbindin gene increases during the ovulatory cycle at a time coincident with eggshell calcification, there is little or no change in the tissue levels of calbindin protein, indicating post-translational control of calbindin levels [4]. Calcium secretion from the shell gland cells increases approximately 7 h after ovulation and reaches a maximum level as the shell is being formed [5]. The hormonal signals affecting changes in the rate of calcium secretion are not fully understood, although estrogen involvement has been suggested [7]. It is suggested that secretion of calcium from the shell gland cells may occur both by active transport and diffusion [8], involving expenditure of metabolic energy [5]. Calcium secretion appears to be linked functionally to luminal HCO_3_ concentration [8]. It seems that there is the involvement of a number of synthetic pathways in eggshell formation. About 37 ion transport genes have been shown to be involved in eggshell formation [9].

The organic components of the eggshell are shell membranes, mammillary cores, shell matrix, cuticle and pigment [10, 11]. The inorganic components of the eggshell are mammillary layer, palisade layer and surface crystal layer [10, 12]. More than 500 eggshell proteins have been identified in laying hens [13, 14]. All layers except for the shell membranes are formed in the shell gland. The membranes are composed of 10% collagen (types I, V and X) as well as 70–75% of other proteins and glycoprotein containing hexosamine and galactose [15–19] and lipids [20, 21]. The mammillary cores contain protein, carbohydrate and fat [22]. The eggshell matrix is a series of layers of protein and acid mucopolysaccharide [10, 23]. Some of the vital eggshell matrix proteins are ovocalyxin-36 [24, 25], ovocleidin-17 [26], ovocalyxin-32 [27] and ovocalyxin-25 [28] all of which possess antimicrobial functions. The cuticle is composed of glycoprotein (90%), polysaccharides (4%), lipids (3%) and inorganic phosphorus (3%) including hydroxyapatite crystals [10, 19, 29]. The major pigments of avian eggshells are protoporphyrin, zinc porphyrin, biliverdin and zinc biliverdin [30]. The calcified eggshell consists primarily of calcite, the most stable polymorph of calcium carbonate [10]. The metallo-proteinase family of proteins has been shown to play a role in reproductive tract remodelling [31]. Genes such as *SPP1* (Secreted phosphoprotein 1), *ACP1* (Acid phosphatase 1), *PENK* (Proenkephalin), *RCAN1* (Regulator of calcineurin 1), *CALB1* and *CYP26A1* (Cytochrome P450 family 26 subfamily A member 1) have been shown to be actively involved in eggshell formation [32, 33]. However, global gene regulation in the eggshell formation of laying hens has not been reported.

We hypothesised that the regulation of genes involved in eggshell formation in the shell gland differs at different stages of eggshell formation on a global scale depending on the shell gland’s molecular and energetic requirements. To test this hypothesis we collected shell gland tissue for analysis when the egg was either forming in the distal magnum or isthmus and in the shell gland regions of the oviduct in brown-egg laying hens. Therefore, the main objective of the current study was to acquire a comprehensive picture of the transcriptional changes in the shell gland of brown-egg laying hens at two different time-points of eggshell formation. We also aimed to identify unknown candidate genes involved in eggshell formation.

## Methods

### Rearing of laying hens

Day old Isa-Brown laying chickens were obtained from the Baiada Hatchery at Tamworth, NSW, Australia. At the hatchery, day-old chickens received Rispens vaccine against Marek’s disease. The chickens were raised in isolation sheds at the University of New England under strict biosecurity protocols. All chickens were fed commercial starter to 4 weeks of age, pullet grower to 18 weeks of age and layer mash until the termination of the experiment. From the isolation sheds, pullets were moved at 18 weeks of age to individual cages in an isolated poultry house. At 35 weeks of age, eggs were collected and processed for traditional egg quality measurements following the method of Samiullah et al. [34]. Hens were then divided into a 1 × 2 factorial design in such a way that the egg weight and eggshell colour (L*) were not significantly different (*P* > 0.05) between the groups (Additional file 1: Table S1). Individual hen oviposition times were recorded by video camera, and each hen was processed at a specific post-oviposition time (5 and 15 h). At the time of euthanasia, the egg in individual hens was either in the distal magnum/isthmus (5 h post-oviposition time-point) or in the shell gland (15 h time-point).

### Tissue collection

A total of forty hens were euthanised with CO_2_ gas and the shell gland was aseptically retracted through an abdominal incision. An approximately 500 mg sample tissue was cut from the centre of the shell gland and transferred directly to RNALater (Sigma Aldrich, Sydney, Australia). The samples were stored at − 20 °C and were processed for total RNA extraction within one day of collection. For total RNA extraction, a whole piece of shell gland tissue (all tissue layers) was processed.

### Total RNA extraction and purification

**Fig. 1:**
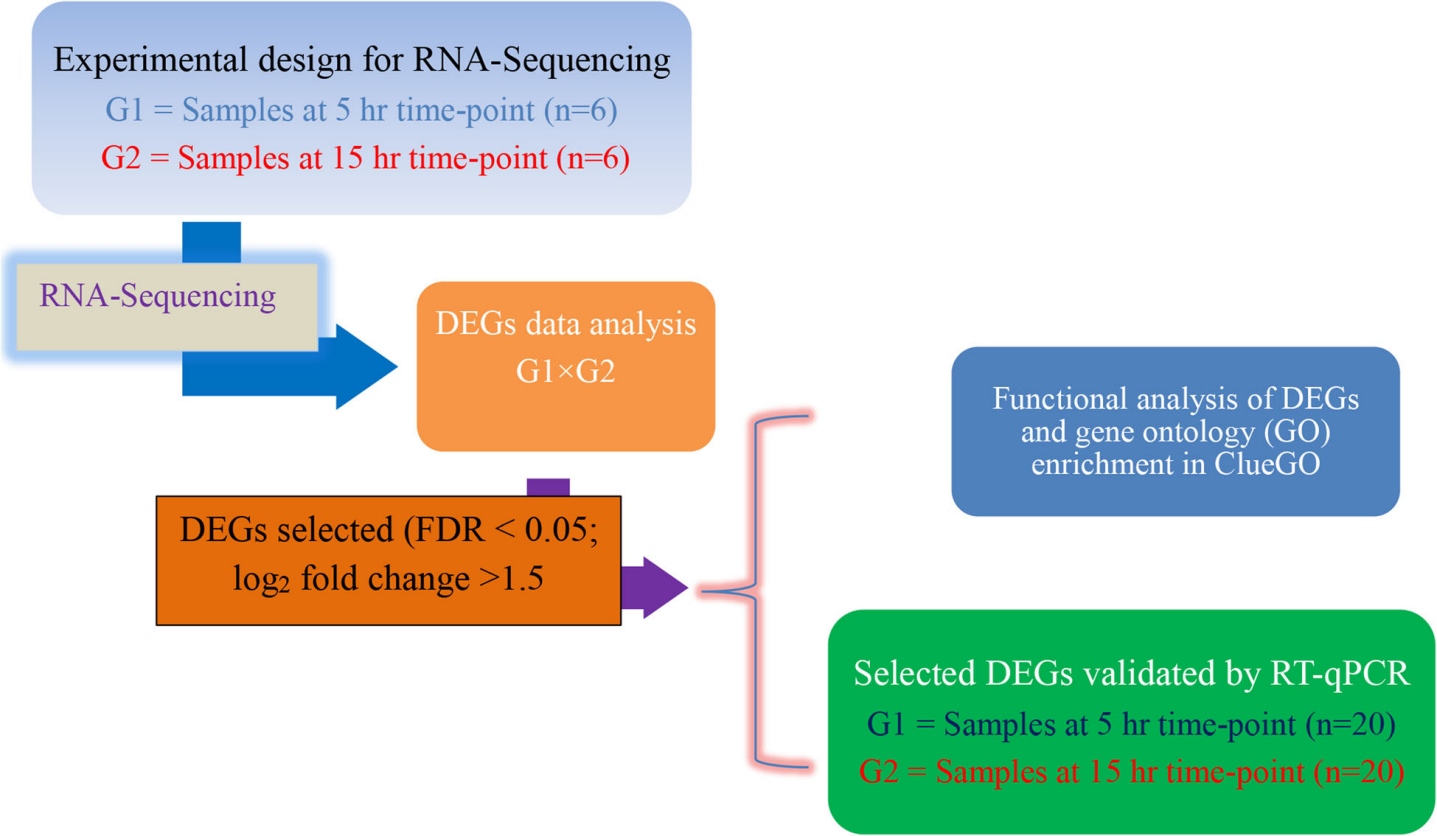
Schematic diagram explaining the selection of samples for RNA-Seq and differentially expressed genes (DEGs) data analysis. To validate the results of RNA-Seq, qPCR was performed on all 40 RNA samples and the data were analysed in qbase+ software for gene expression study. For bioinformatics analysis, the 5 h time-point was taken as the reference control as compared to the 15 h time-point

Total RNA was extracted using TRIsure (Bioline, Australia), according to the manufacturer’s instructions. Briefly, an approximately 100 mg of tissue (wet weight) was homogenized in 1 mL of TRIsure using an IKA T10 basic Homogenizer (Wilmington, NC, USA). After the RNA pellets were washed with 1.5 mL ethanol (75%), 50 μL of UltraPure™ DEPC-treated water (Ambion, USA) was used to dissolve RNA pellets. The dissolved RNA was further purified using an RNeasy Mini Kit (Qiagen, GmbH, Hilden, Germany) as per the manufacturer’s instructions. A DNase-I step was performed to remove traces of genomic DNA from the extracted total RNA. The elution of RNA from the spin column with 50 μL of RNase-free water was repeated twice and the eluted RNA solutions were mixed thoroughly. The purified RNA was analysed in a NANODROP-8000 spectrophotometer (ThermoFisher Scientific, Wilmington, DE, USA) to measure its quantity and purity. The absorbance measurements of the spectrophotometer 260/280 and 260/230 ratios were in the range of 1.8–2.1 and 1.9–2.2, respectively. RNA integrity and purity were further examined in an Agilent 2100 Bioanalyzer (Agilent Technologies, Waldbronn, Germany) as per the manufacturer’s instructions for an Agilent RNA 6000 Nano Kit. All the RNA showed distinct 18S and 28S bands with an average RNA integrity number (RIN) of > 9.1. Representative shell gland purified total RNA samples (Fig. 1) were processed by the Australian Genome Research Facility (AGRF) for RNA-Seq analysis.

### cDNA libraries preparation

Illumina’s TruSeq Stranded mRNA Prep Kit was used for processing the RNA samples. The process included mRNA purification via oligo (dT) beads, fragmentation of mRNA with divalent cations and heat, and 1st strand cDNA and 2nd strand cDNA syntheses. cDNA libraries were prepared by DNA fragment end repair, 3′ adenylation of DNA fragments, sequence adaptor ligation and amplification of library via PCR. In total, 12 cDNA libraries (i.e. one library for each sample) were constructed for sequencing - 6 samples for each of the time-points at 5 h and 15 h post-oviposition. Sequencing of libraries using 100 bp single read was performed on an Illumina HiSeq 2000 sequencing system.

### Sequence quality control and sequence data evaluation

The primary sequence data were generated using the Illumina bcl2fastq 2.17.1.14 pipeline.

Initial quality control of the RNA sequences was evaluated by FastQC v0.11.5 [35]. The raw reads were also screened for the presence of any Illumina adaptor/overrepresented sequences, low quality sequences, empty reads and cross species contamination. Illumina adaptors and contaminated sequences were removed through trim_galore and Fastq_clean (https://ieeexplore.ieee.org/document/6999309/).

### Reads mapping and raw gene counts

Tophat aligner (v2.0.14) [36] was used to map reads to the genomic sequences. The counts of reads mapping to each known gene were summarised at gene level using the featureCounts v1.4.6-p5 utility of the subread package (http://subread.sourceforge.net/). The cleaned sequence reads were aligned against the *Gallus gallus* genome (Built version 5 Ensembl release 86) [37].

### Reference guided transcript assembly

The transcripts were assembled with Stringtie tool v1.2.4 (http://ccb.jhu.edu/software/stringtie/) utilising the reads alignment and reference annotation based assembly option (RABT). This option generated assembly for known and potentially novel transcripts. The Ensembl annotations (Gallus_gallus.Gallus_gallus-5.0.86.gtf) for genome were used as a guide. Common gene names were converted to Entrez IDs using Ensembl of chicken genome assembly.

### Differential gene expression data analysis

Gene expression was calculated in counts-per-million (CPM) with a hard filter of 0.5 in edgeR (v3.14.0). Trimmed mean of M values (TMM) normalisation was applied to estimate gene expression and identify differentially expressed genes (DEGs) using R packages (R version 3.3.1) ‘edgeR’ [38] and ‘limma’ (3.28.21) [39]. During differential gene data analysis, false discovery rate/adjusted *p*-value was used for multiple test comparison (BH-adjustment). DEGs obtained at the 15 h time-point were compared to the 5 h time-point using the later as reference. To obtain further insight on the functions of the DEGs encoding hypothetical proteins, Ensembl BLAST/BLAT searches were performed with nucleotide and protein sequences queries using a cut-off e-value of < 10^− 20^.

### Hierarchical clustering analysis

Average gene counts for the top 50 significantly down- or up-regulated genes at the 15 h relative to the 5 h time-point of eggshell formation were considered when performing the hierarchical clustering. The clustering was performed in gplots (version 3.0.1) of R packages (version 3.3.1), and the results were presented as heatmaps.

### Functional annotation of DEGs

The DEGs (log_2_ fold change > 1.5; FDR < 0.05) were subjected to functional analysis using ClueGO version 2.2.6 [40, 41] + CluePedia version 1.2.6 [42] plugins in Cytoscape version 3.4.0 [43] as has previously been used in a similar study [44]. The DEGs were enriched for terms specific for biological process (BP), molecular functions (MF) and cellular component (CC). The annotation enrichment of the DEGs was performed with the 5 h time-point being considered as a reference control.

To create the annotation network, ClueGO investigates the distribution of the target genes across the Gene Ontology (GO) terms and pathways. CluePedia is a Cytoscape plugin for pathway insights using integrated experimental and in silico data [42]. CluePedia extends the functionality of ClueGO down to gene level [42]. In ClueGO analysis, the *P* value was calculated using the right-sided hypergeometric tests with Benjamini-Hochberg adjustment for multiple test correction [45]. An adjusted *P* ≤ 0.001 indicated a statistically significant deviation from the expected distribution, and that the corresponding GO terms and pathways were enriched for the target genes. The association strength between the terms was calculated using a corrected kappa statistic score of 0.4, in ClueGO [41, 46]. The relationship between the selected terms was defined based on their shared genes in a similar way. The created network showed the GO terms as nodes and size of the nodes reflected the enrichment significance. The network was automatically laid out using the organic layout algorithm supported by the Cytoscape software [43]. Functional groups represented by their most significant term were visualized in the network providing an insightful view of their interrelations [40].

### Primer design, specificity and amplification efficiency for qPCR

Primers for the candidate target genes were designed in NCBI software by choosing an option for exon-intron spanning (Table 1). Primers for the reference and *CALB1* genes were sourced from published literature. Specific amplifications of the primers were confirmed by a single peak of melting curve analysis and a single amplicon band of appropriate size using Agilent 2100 Bioanalyzer gel electrophoresis and a DNA 1000 Kit (Agilent Technologies, Waldbronn, Germany). PCR amplification efficiencies and correlation coefficients (R^2^) were determined with the amplifications of a series of six 10-fold RNA dilutions [47–49]. Amplification efficiency was calculated based on the following equation: $E\ \left(\%\right)=\left({10}^{\frac{1}{- slope}}-1\right)\times 100$ %. The primer pairs were used for qPCR analysis only if the amplification efficiency was in a range of 90 to 105%, and linear correlation coefficient R^2^ > 0.980 [49–51].

**Table 1:** Candidate target and reference* genes in expression studies by qPCR in the shell gland of laying hens

Gene Name	Gene Symbol	Primer sequence (5′-3′)	Amplicon size (bp)	Ta °C	PCR Efficiency (%)	Accession No.	Reference
Secreted phosphoprotein 1	*SPP1*	F-CCAGGAAGCTCATTGAGGATG R-GCTGTCGTTCTCTACGCTCT	134	60	101	NM_204535.4	This study
Proopiomelanocortin	*POMC*	F-TGGTGTTTTGGCGTGTGC R-CATCACGTACTTGCGGATGC	115	65	105	NM_001031098.1	This study
Calbindin	*CALB1*	F: TTGGCACTGAAATCCCACTGA R: CATGCCAAGACCAAGGCTGA	116	60	100	NM_205513.1	[71]
Claudin 16	*CLDN16*	F- TACCTTGCTCATTGCAGGTCT R- GTGAGCAGGGACCCAGATAAG	186	63	105	XM_426702.3	This study
G protein subunit gamma 4	*GNG4*	F- CAGACCAATGCACAAGTTTCA R- GCCTCAAGTGGAAAGGTCAC	243	63	97	XM_004935468.2	This study
Potassium voltage-gated channel subfamily H member 1	*KCNH1*	F- AGAGGCAGAGATCCAGACGA R- GGTCTGATGTCCCAGACGTT	160	63	93	XM_015283863.1	This study
BPI fold containing family B, member 3	*BPIFB3*	F- CCATGCAACAAGTGCTGTCC R- AGCAGTTGCCACTGAGATCC	234	63	94	NM_001030861	This study
Rho related BTB domain containing 3	*RHOBTB3*	F- GACGTCGCATCTGTGATCC R- TCTTCCTTAGCTCGGCGTTA	171	63	95	ENSGALG00000014675	This study
Somatostatin II	*SS2*	F- GCTCTTGGAGAGCTCAGACG R- GCACTAGCAGGAGGTGAAGG	160	65	97	NM_204455.1	This study
Otopetrin 2	*OTOP2*	F- GGAGCAAGCAATTGCCCAAA R- CGCTGCTTTGCTGCCTG	202	63	99	XM_003642368.3	This study
Klotho beta	*KLB*	F- GAGCAATACGGGGGATGGAA R- GCATGAGCCTTGATCAGATTGT	224	61	101	XM_003641245.3	This study
Glycoprotein hormones, alpha polypeptide	*CGA*	F- GTCCAGAGTGCAAGCTAGGG R- GCTACACAGCACGTTGCTTC	166	63	105	:NM_001278021	This study
GATA-binding factor 3	*GATA3*	F- CAGAAGGCAGGGAGTGTGTAA R- GCTGCAGACAGCCTTCTCT	157	63	100	NM_001008444	This study
TYRO3 protein tyrosine kinase	*TYRO3*	F- GTGCAGTGCAGCAATGAGAT R- CAGCCTGTATCCCAGGACAT	186	61	98	NM_204627	This study
Matrix metallopeptidase 13	*MMP13*	F- AGCTCTATGGTGCTGGAGAC R- CCTGTCCTTGAAGACCAGCAT	128	63	100	NM_001293090	This study
Gap junction protein, alpha 8	*GJA8*	F- CTGCTATGATGAGGCCTTCC R- GGCAGCTTCTCTTCATCCAC	186	63	98	NM_204997.1	This study
Carbonic anhydrase 9	*CA9*	F- CTCAGTGACAGCAGCAGGA R- TGGCAAAGAGCACTCCGAAT	80	63	99	XM_004937157.2	This study
Cytochrome P450 family 7 subfamily A member 1	*CYP7A1*	F- AGGAGGCAATGAGGCTATCG R- TGAGTGTCAAGGGATCAGCA	171	63	96	NM_001001753.1	This study
Galactose-3-O-sulfotransferase	*GAL3ST2*	F- TGCTTCGAGGACTACCAAAAA R- TGGGTCTTGAGGAACATGACG	177	61	96	NM_001277431.1	This study
TATA-Box Binding Protein	TBP*	F: TAGCCCGATGATGCCGTAT R: GTTCCCTGTGTCGCTTGC	147	61	97	NM_205103	[72]
Tyrosine 3-monooxygenase/tryptophan 5-monooxygenase activation protein, zeta polypeptide	YWHAZ*	F- TTGCTGCTGGAGATGACAAG R- CTTCTTGATACGCCTGTTG	61	60	104	NM_001031343.1	[73]

The candidate target genes were selected from the DEGs either down- or up-regulated at the 15 h relative to the 5 h time-point of eggshell formation

### Quantitative PCR validation of RNA-Seq results

Quantitative PCR was performed on 40 samples of shell gland tissue RNA with the SensiFAST SYBR® Lo-ROX One-Step RT-PCR Kit (Bioline, Sydney, Australia). Master mix was prepared as per the manufacturer’s protocol and 4 μL of RNA template with 1:100 dilutions was added to the reaction wells using a QIAgility robotic (Qiagen, Hilden, Germany). The reaction was run in duplicates of 20 μL in a Rotor-gene Disc 100 (Qiagen, Hilden, Germany) with a Rotor-Gene Q thermocycler (Qiagen, Hilden, Germany). No template control (NTC) and no reverse transcriptase (−RT) control were also included to detect possible contamination. Thermocycling conditions for a 2-step PCR were: reverse transcription at 45 °C for 10 min, first denaturation at 95 °C for 2 min, then 40 cycles of denaturation at 95 °C for 5 s and annealing at appropriate temperatures (shown in Table 1) for 20s. The fluorescent data were acquired at the end of each annealing step during PCR cycles. A melting step was conducted to assess the specificity of PCR amplification.

### Statistical analysis

Egg quality data were analysed by Statview software (SAS Institute Inc., Version 5.0.1.0). To calculate the relative expression of the candidate target genes, Cq values were analysed in qbase+ software version 3.0 [52]. The Cq values of target genes were normalized against previously optimised reference genes (*TBP*: TATA-Box binding protein and *YWHAZ*: Tyrosine 3-monooxygenase/Tryptophan 5-monooxygenase activation protein zeta) [53] to obtain normalized relative quantities of individual genes. Candidate target gene specific amplification efficiencies were used based on the method of Pfaffl [54]. The normalized relative quantities were further analysed in Statview software to compare the means from the time-points of 5 and 15 h. Significant differences were separated by the Tukey-Kramer test at probability < 0.05.

## Results

### Differential gene expression in shell gland tissue

A total of 261,684,549 (26.17 Gb of data bulk) clean reads with an average length of 100 bp were generated from the twelve libraries divided into two groups (G1 and G2; Fig. 1). The reads feature summary is depicted in Table 2. The feature summary shows that the percentage of reads mapped to *Gallus gallus* genome was ≥80%. Multi-dimensional scaling (MDS) plot showed that there was a significant effect of time-point on the expressed genes (Additional file 2: Figure S1).

**Table 2:** Sequence quality and alignment information of 12 shell gland samples in two groups (G1 and G2)

Sample name	Total reads	Number of reads mapped to chicken genome	Percentage of reads mapped to chicken genome	Number of reads mapped to one feature	Percentage of reads mapped to one feature	Number of mapped reads not mapped to any feature	Percentage of total reads that mapped to the genome but not to any known features
G1a	23,419,963	18,942,266	80.88%	12,137,770	51.83%	5,855,006	25.00%
G1b	22,633,784	18,410,799	81.34%	11,944,759	52.77%	5,541,424	24.48%
G1c	22,478,580	18,055,302	80.32%	11,646,051	51.81%	5,503,560	24.48%
G1d	21,759,465	17,607,983	80.92%	11,461,812	52.68%	5,268,439	24.21%
G1e	22,657,131	18,480,220	81.56%	12,031,069	53.10%	5,536,020	24.43%
G1f	21,832,656	17,675,184	80.96%	11,615,956	53.20%	5,182,041	23.74%
G2a	21,033,600	17,095,487	81.28%	11,393,823	54.17%	4,839,852	23.01%
G2b	20,845,746	16,707,543	80.15%	11,030,858	52.92%	4,836,757	23.20%
G2c	20,784,865	16,624,745	79.98%	11,034,132	53.09%	4,745,972	22.83%
G2d	21,073,856	17,140,851	81.34%	11,265,366	53.46%	5,021,572	23.83%
G2e	21,309,212	17,398,557	81.65%	11,516,521	54.04%	5,014,414	23.53%
G2f	21,828,196	17,648,497	80.85%	11,763,767	53.89%	4,986,438	22.84%

There were 6 shell gland samples (a-f) in each individual group. G1 and G2 refer to shell gland tissue samples obtained at 5 and 15 h time-points of eggshell formation, respectively

A total of 14,334 gene transcripts were assessed for differential expression after filtering was applied. Differential gene expression analysis showed 691 (log_2_ fold change > 1.5; FDR < 0.05) differentially expressed genes (DEGs) between the 5 h time-point and the 15 h time-point of eggshell formation. Among the 691 DEGs, there were 278 significantly down-regulated and 413 significantly up-regulated genes at the 15 h time-point relative to the 5 h time-point of eggshell formation. Among the DEGs at the 15 h relative to the 5 h time-point, *SLC13A5* (Solute carrier family 13 member 5), *KLB* (Klotho beta), *XAF1* (XIAP associated factor 1), *FIBIN* (Fin bud initiation factor homolog (zebrafish)) and *POMGNT1* (Protein O-linked mannose N-acetylglucosaminyltransferase 1 (Beta 1,2-) were the top five most down-regulated genes. A full list of the top 50 significantly down-regulated genes at the 15 h relative to the 5 h time-point is shown in Table 3. Among the DEGs that were significantly up-regulated at the 15 h relative to the 5 h time-point, the top five genes were *POMC* (Proopiomelanocortin5), *CALB1* (Calbindin), *SPP1* (secreted phosphoprotein 1), *NEU4* (Neuraminidase 4) and *CEMIP* (Cell migration inducing hyaluronan binding protein). A full list of the top 50 significantly up-regulated genes at the 15 h relative to the 5 h time-point is shown in Table 4.

**Table 3:** Top 50 down-regulated DEGs at 15 h relative to 5 h time-point

Gene symbol	Gene name	Fold change	FDR
*SLC13A5*	Solute carrier family 13 member 5	−6.516	1.99E-07
*KLB*	Klotho beta	−5.998	0.00079
*XAF1*	XIAP associated factor 1	−5.316	9.26E-05
*FIBIN*	Fin bud initiation factor homolog (zebrafish)	−4.894	1.40E-07
*POMGNT1*	Protein O-linked mannose N-acetylglucosaminyltransferase 1 (Beta 1,2-)	−4.828	0.00026
*MMP13*	Matrix metallopeptidase 13	−4.673	0.00089
*CTNNA3*	Catenin alpha 3	−4.625	0.00239
*GJA8*	Gap junction protein alpha 8	−4.522	0.00089
*CA9*	Carbonic anhydrase 9	−4.519	0.00042
*HABP2*	Hyaluronan binding protein 2	−4.464	0.00119
*ARHGAP25*	Rho GTPase-activating protein 25	−4.284	1.18E-06
*SEMA3G*	Semaphorin 3G	−4.254	3.39E-06
*fibrinogen*	Fibrinogen beta chain	−4.199	0.00057
*CYP7A1*	Cytochrome P450 family 7 subfamily A member 1	−4.170	0.00081
*ADPRHL1*	ADP-ribosylhydrolase like 1	− 4.103	0.00035
*GHRHR*	Growth hormone releasing hormone receptor	−4.075	0.0037
*TCERG1L*	Transcription elongation regulator 1 like	−3.925	4.38E-05
*FREM2*	FRAS1 related extracellular matrix protein 2	− 3.835	0.00608
*NR1D1*	Nuclear receptor subfamily 1, group D, member 1	−3.785	0.00235
*EVPL*	Envoplakin	−3.749	1.20E-05
*ODZ1*	Teneurin transmembrane protein 1	−3.732	0.00339
*TDT*	DNA nucleotidylexotransferase	3.714	0.00241
*SAMD7*	Sterile alpha motif domain containing 7	−3.620	0.00405
*HAS2*	Hyaluronan synthase 2	−3.564	0.00034
*PCBP2*	Poly (RC) binding protein 2	−3.548	0.00125
*SLC25A15*	Solute carrier family 25 (mitochondrial carrier; ornithine transporter) member 15	−3.545	4.48E-05
*ADRA2A*	Adrenoceptor alpha 2A	−3.512	0.00042
*CBX2*	Chromobox 2	−3.484	0.00497
*TNFSF10*	Tumor necrosis factor superfamily member 10	−3.475	5.95E-05
*SLC26A4*	Solute carrier family 26 member 4	−3.467	0.03297
*NTN1*	Netrin 1	−3.449	0.01807
*VWD*	Von Willebrand Factor	−3.448	0.00591
*PLA2G4E*	Cytosolic phospholipase A2 epsilon-like	−3.409	0.03367
*DAB1*	DAB1, reelin adaptor protein	−3.383	0.00025
*FAM159A*	Family with sequence similarity 159 member A	−3.347	0.00295
*AMER2*	APC membrane recruitment protein 2	− 3.299	0.00011
*GRXCR1*	Glutaredoxin and cysteine rich domain containing 1	−3.205	0.05005
*BCAS1*	Breast carcinoma amplified sequence 1	− 3.197	0.0048
*SLC13A2*	Solute carrier family 13 member 2	−3.162	2.00E-05
*NF2L*	Neurofibromin 2 (merlin)-like	−3.111	0.00156
*CGA*	Glycoprotein hormones, alpha polypeptide	−3.078	0.01157
*C8B*	Complement C8 beta chain	−3.063	0.01559
*SYT15*	Synaptotagmin 15	−3.055	0.00329
*RASD1*	Ras related dexamethasone induced 1	−3.047	5.25E-06
*KCNA1*	Potassium voltage-gated channel subfamily A member 1	−3.045	0.01937
*GATA3*	GATA-binding factor 3	−3.036	1.18E-06
*PLA2G4F*	Phospholipase A2 group IVF	−3.029	0.03613
*SRL*	Sarcalumenin	−2.959	0.00252
*DSC1*	Desmocollin 2	−2.954	0.00012
*ANO3*	Anoctamin-3 isoform 1	− 2.933	0.00307

Fold change (FC) was calculated in log_2_ value. Minus (−) sign shows down-regulation of the genes at the 15 h relative to the 5 h time-point

**Table 4:** Top 50 up-regulated DEGs at 15 h relative to 5 h time-point

Gene symbol	Gene name	Fold change	FDR
*POMC*	Proopiomelanocortin	+ 9.179	0.0005
*CALB1*	Calbindin	+ 8.081	3E-08
*SPP1*	Secreted phosphoprotein 1	+ 7.993	2E-07
*NEU4*	Neuraminidase 4	+ 7.682	0.0021
*CEMIP*	Cell migration inducing hyaluronan binding protein	+ 7.555	9E-06
*GAL3ST2*	Galactose-3-O-sulfotransferase 2	+ 6.999	0.0005
*SLC6A17*	Solute carrier family 6 member 17	+ 6.643	0.0271
*GNG4*	G protein subunit gamma 4	+ 6.586	0.0416
*BPIFB3*	BPI fold containing family B, member 3	+ 6.414	3E-06
*ECEL1*	Endothelin converting enzyme Like 1	+ 6.408	0.0312
*REG4*	Regenerating family member 4	+ 6.272	0.0017
*ANGPTL3*	Angiopoietin like 3	+ 6.199	0.0002
*LOC415478*	Transmembrane protein 2-like	+ 6.062	6E-06
*KCNH1*	Potassium voltage-gated channel, subfamily H (eag-related), member 1	+ 5.956	0.0009
*GNRHR*	gonadotropin-releasing hormone receptor	+ 5.949	0.0122
*MKI67*	Marker of proliferation Ki-67	+ 5.815	0.0127
*BPIL3*	Bactericidal/permeability-increasing protein-like 3	+ 5.250	0.0004
*SLC29A4*	Solute carrier family 29 member 4	+ 5.183	8E-05
*WNT11*	Wnt family member 11	+ 4.799	4E-05
*CHRD*	Chordin	+ 4.691	3E-05
*OFCC1*	Orofacial cleft 1 candidate gene 1 protein homolog	+ 4.330	0.0005
*GPR183*	G Protein-coupled receptor 183	+ 4.329	0.0001
*ETV4*	ETS variant 4	+ 4.309	0.0264
*RHOBTB3*	Rho related BTB domain containing 3	+ 4.305	0.0036
*OTOP2*	Otopetrin 2	+ 4.226	1E-05
*MFSD13A*	Major facilitator superfamily domain containing 13A	+ 4.190	2E-08
*TNFRSF6B*	TNF receptor superfamily member 6b	+ 4.182	0.0005
*PLPPR4*	Phospholipid phosphatase related 4	+ 4.161	8E-05
*B3GNT7*	BetaGal beta-1,3-N-acetylglucosaminyltransferase 7	+ 4.139	9E-05
*SEMA3D*	Semaphorin 3D	+ 4.126	0.0206
*FAM163A*	Family with sequence similarity 163 member A	+ 4.125	0.004
*RUBCNL*	RUN and cysteine rich domain containing beclin 1 interacting protein like	+ 4.049	1E-06
*OTOP3*	Otopetrin 3	+ 4.003	0.0014
*BHLHA15*	Basic helix-loop-helix family member a15	+ 3.998	0.0354
*SLC38A8*	Solute carrier family 38 member 8	+ 3.941	0.0013
*TUBB3*	Tubulin beta 3 class III	+ 3.924	0.0002
*ETNK1*	Ethanolamine kinase 1	+ 3.918	4E-06
*MIR6556*	Gga-mir-6556	+ 3.913	0.0017
*PERM1*	PPARGC1 and ESRR induced regulator, muscle 1	+ 3.902	0.007
*COL21A1*	Collagen type XXI alpha 1 chain	+ 3.885	0.0003
*NPTX1*	Neuronal pentraxin 1	+ 3.884	0.0106
*EREG*	Epiregulin	+ 3.867	0.0434
*FABP3*	Fatty acid binding protein 3	+ 3.824	4E-05
*SS2*	Somatostatin II	+ 3.804	0.0062
*PHGDH*	Phosphoglycerate dehydrogenase	+ 3.777	0.0004
*HEPACAM*	Hepatic and glial cell adhesion molecule	+ 3.751	0.0055
*MAP 3 K15*	Mitogen-activated protein kinase kinase kinase 15	+ 3.737	1E-05
*WSCD2*	WSC domain containing 2	+ 3.716	6E-07
*NKAIN1*	Na+/K+ transporting ATPase interacting 1	+ 3.694	0.0038
*PTN*	Pleiotrophin	+ 3.693	0.0016

Fold change (FC) was calculated in log_2_ value. Plus (+) sign shows up-regulation of the genes at 15 h relative to 5 h time-point

### DEGs analysis for hypothetical functions

Most of the DEGs with hypothetical functions appeared to possess domains that function in diverse cellular activities (Table 5). The associated GO terms showed that the functions of the unknown genes may be correlated with the synthesis and secretory activities in the shell gland during an eggshell formation. In addition, there were 6.11 and 6.31% of lincRNA significantly (log_2_ fold change > 1.5; FDR < 0.05) down- and up-regulated, respectively, at the 15 h relative to the 5 h time-point of eggshell formation.

**Table 5:** Putative functions of mRNA sequences that did not annotate to any known gene IDs during Ensembl annotations of RNA-Seq transcripts

Group	Sequence ID	Gene ID	Associated GO term	Fold change	FDR
^a^G1	ENSGALG00000039411	*COL25A1*	Heparin binding and beta-amyloid binding	− 4.899	0.0031
	ENSGALG00000032113	*NAT8L*	N-acetyltransferase activity and aspartate N-acetyltransferase activity	− 4.038	9E-05
	ENSGALG00000029321	*NAT8L*	N-acetyltransferase activity and aspartate N-acetyltransferase activity	− 3.857	0.0009
	ENSGALG00000030322	*TMEM163*	No associated GO term found	− 3.349	0.0003
	ENSGALG00000038759	*PARD6B*	No associated GO term found	− 2.867	0.0007
^b^G2	ENSGALG00000037163	*SLC6A4*	Protein homodimerization activity and Rab GTPase binding	+ 6.402	0.0004
	ENSGALG00000006393	*ADGRG6*	G-protein coupled receptor activity and transmembrane signaling receptor activity	+ 5.243	0.0306
	ENSGALG00000039812	*GPR6*	G-protein coupled receptor activity and sphingosine-1-phosphate receptor activity	+ 5.154	0.0014
	ENSGALG00000029410	*NR1D1*	Transcription factor activity, sequence-specific DNA binding and RNA polymerase II core promoter proximal region sequence-specific DNA binding	+ 4.578	0.0031
	ENSGALG00000041414	*BHLHE41*	Protein homodimerization activity and RNA polymerase II core promoter proximal region sequence-specific DNA binding	+ 4.419	0.0003
	ENSGALG00000042845	*PDE3A*	3,5-cyclic-nucleotide phosphodiesterase activity and 3,5-cyclic-AMP phosphodiesterase activity	+ 3.885	0.0039
	ENSGALG00000035935	*UNC13C*	Diacylglycerol binding	+ 3.701	7E-06
	ENSGALG00000033066	*UBE2E2*	Ligase activity and acid-amino acid ligase activity	+ 3.672	0.0166
	ENSGALG00000033883	*PCDH7*	Calcium ion binding	+ 3.125	0.0042
	ENSGALG00000031565	*ZNF277*	RNA polymerase II core promoter sequence-specific DNA binding	+ 3.005	0.0399
	ENSGALG00000037545	*GRIP2*	Protein C-terminus binding and receptor signaling complex scaffold activity	+ 2.937	0.0009
	ENSGALG00000008047	*TP53I11*	Ligase activity and ubiquitin protein ligase activity	+ 2.903	0.0216
	ENSGALG00000011860	*MYO16*	Actin binding and actin filament binding	+ 2.596	0.0036
	ENSGALG00000042801	*NT5DC4*	Hydrolase activity and 5-nucleotidase activity	+ 2.396	0.0298
	ENSGALG00000039716	*HPCA*	Calcium ion binding and actin binding	+ 2.304	1E-05
	ENSGALG00000041604	*NPTXR*	No associated GO term found	+ 2.295	0.0051
	ENSGALG00000030673	*KCTD14*	NADH dehydrogenase (ubiquinone) activity	+ 2.096	0.0076
	ENSGALG00000042104	*ROBO1*	Identical protein binding and LRR domain binding	+ 2.044	0.0119
	ENSGALG00000038532	*ESPN*	Actin binding and SH3 domain binding	+ 1.878	0.0086
	ENSGALG00000038993	*NEGR1*	No associated GO term found	+ 1.842	3E-06
	ENSGALG00000041238	*SEMA3B*	Receptor activity	+ 1.798	3E-05
	ENSGALG00000042411	*FAM198B*	No associated GO term found	+ 1.765	0.0009
	ENSGALG00000043703	*ELN*	Extracellular matrix structural constituent and extracellular matrix constituent conferring elasticity	+ 1.630	0.0034
	ENSGALG00000043198	*DNPEP*	Metallopeptidase activity and aminopeptidase activity	+ 1.609	0.0021
	ENSGALG00000043209	*ADGRB2*	G-protein coupled receptor activity and transmembrane signaling receptor activity	+ 1.573	0.0267

To retrieve the best homology hit, the sequence IDs were blasted against chicken, duck, turkey and human reference genomes in Ensembl BLAT database. The cut off criterion was established as e-value <10E^− 20^. ^a^Represents genes significantly (log_2_ fold change > 1.5; FDR < 0.05) down-regulated at the 15 h relative to the 5 h time-point. ^b^Represents genes significantly up-regulated at the 15 h relative to the 5 h time-point

### Functional annotation of DEGs down- or up-regulated at 15 h relative to 5 h time-point

**Fig. 2:**
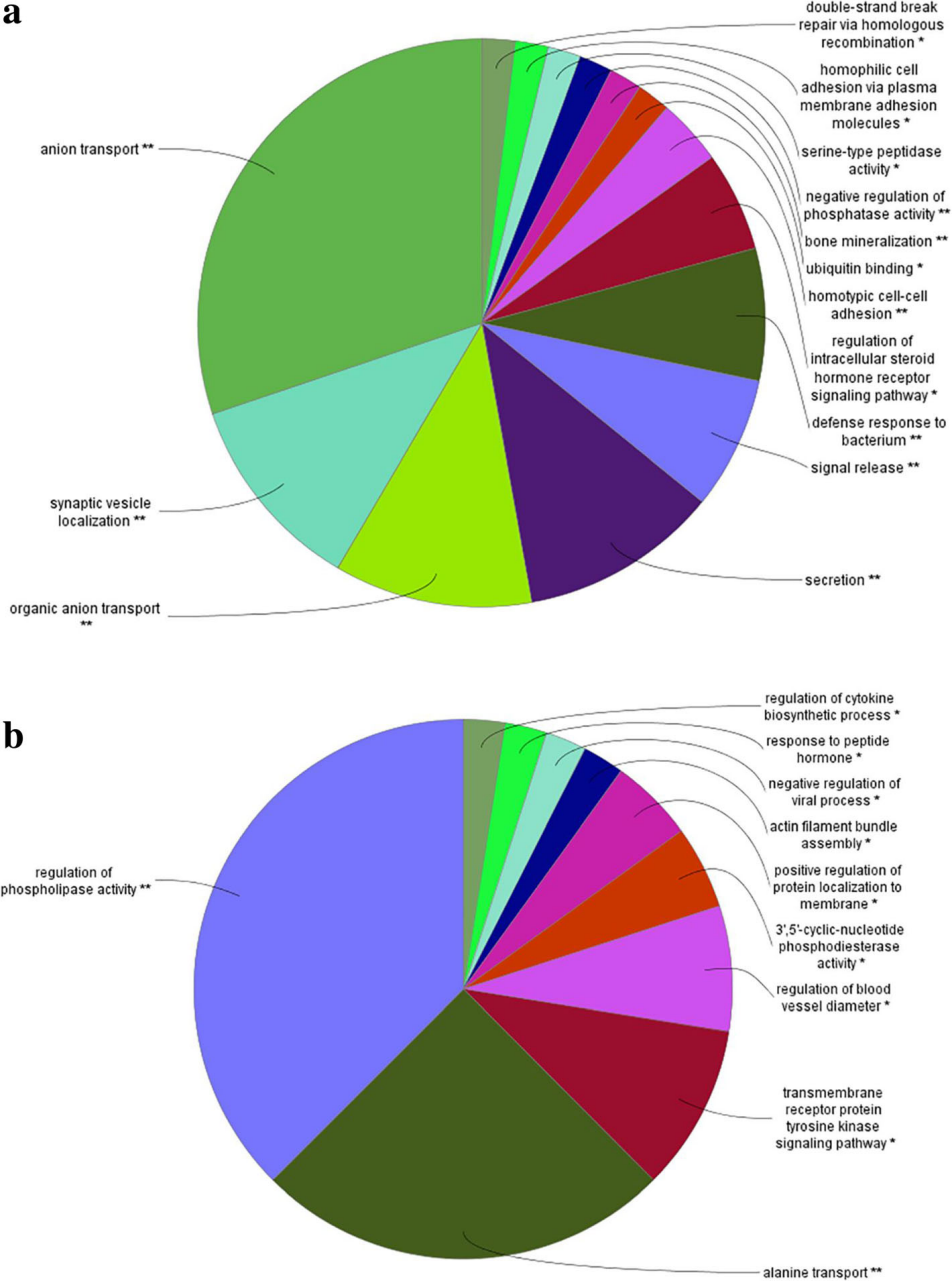
Functional map of DEGs (log_2_ fold change > 1.5; FDR < 0.05) enriched for GO terms specific for biological process, cellular component and molecular function. The chart fragments represent the number of genes associated with the terms as a proportion with the total number of genes within the GO term. **a** GO terms associated with genes significantly down-regulated at the 15 h relative to the 5 h time-point of eggshell formation. **b** GO terms associated with genes significantly up-regulated at the 15 h relative to the 5 h time-point of eggshell formation. **P < 0.001 and *P < 0.01 show the level of significance of the enriched terms

**Fig. 3:**
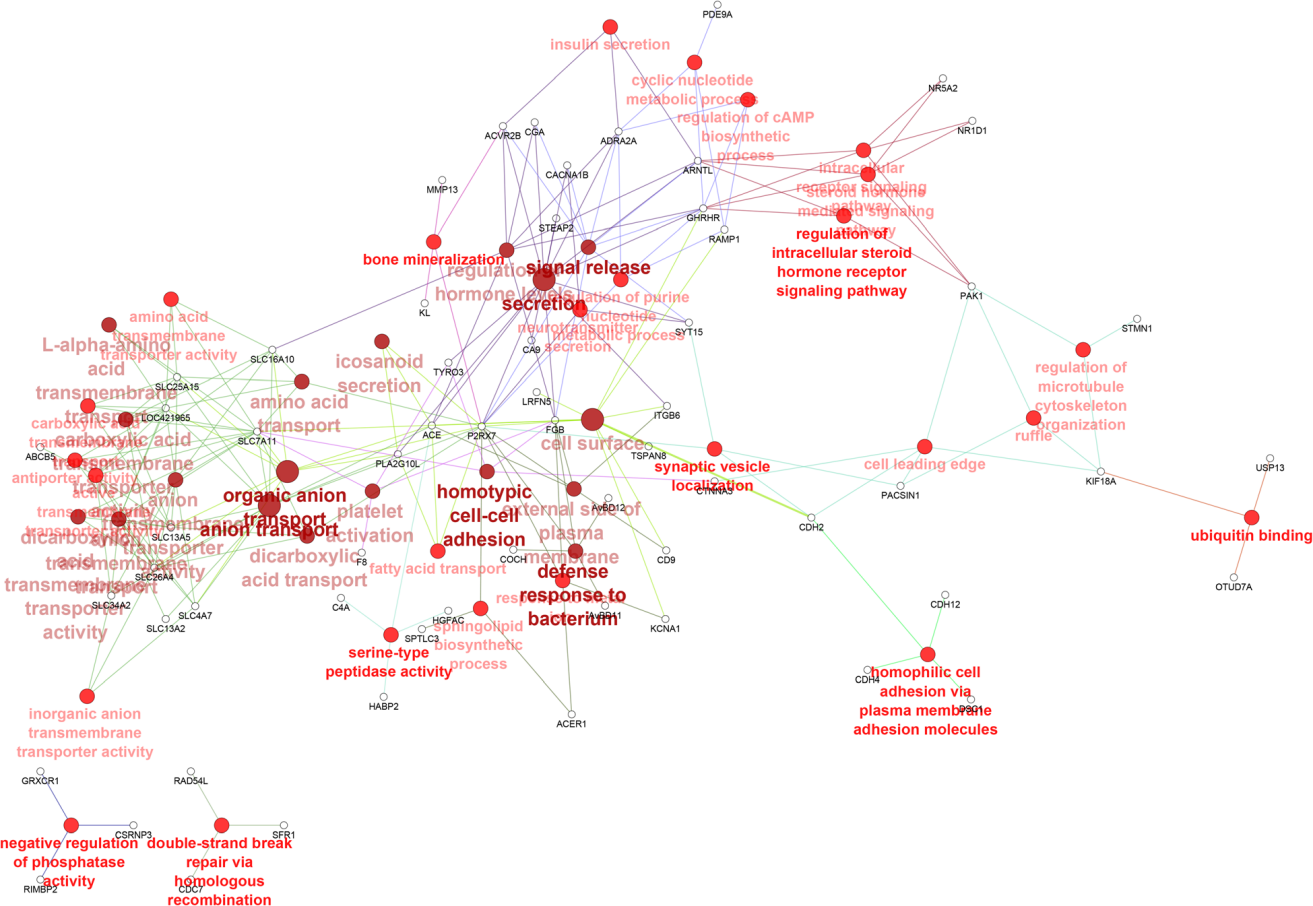
Network representation of the enriched GO terms and their associated genes obtained from the mapping of down-regulated genes at the 15 h relative to the 5 h time-point. The GO terms were identified as nodes and linked based on their *p*-value < 0.05 and kappa score level (> 0.4). Functionally related groups partially overlapped. The terms are labelled in colours according to hierarchical clustering of GO terms. Terms which have not been grouped are shown in grey

**Fig. 4:**
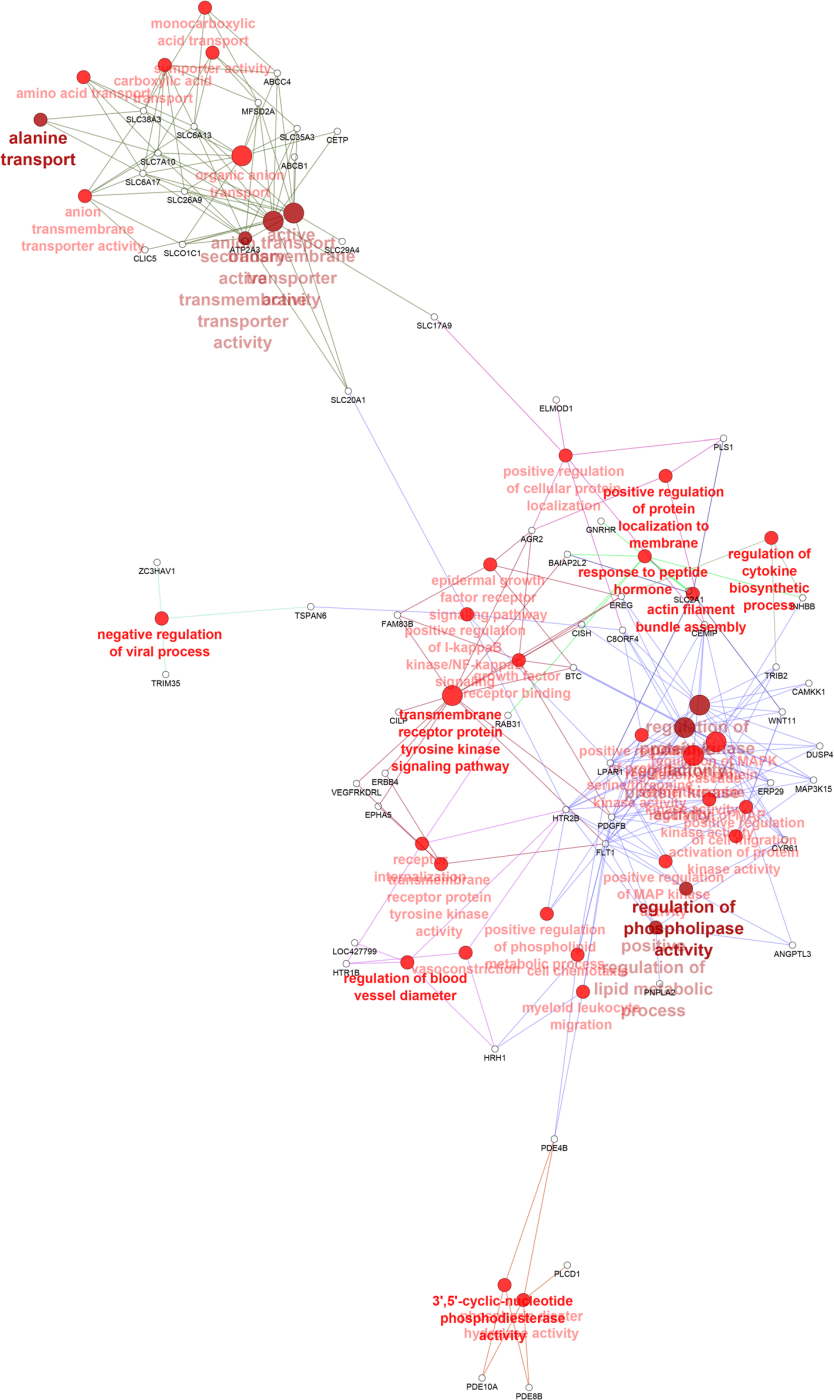
Network representation of the enriched GO terms and their associated genes obtained from the mapping of up-regulated genes at the 15 h relative to the 5 h time-point. The GO terms were identified as nodes and linked based on their p-value < 0.05 and kappa score level (> 0.4). Functionally related groups partially overlapped. The terms are labelled in colours according to hierarchical clustering of GO terms. Terms which have not been grouped are shown in grey

An enrichment gene set analysis was performed to identify the associated Gene Ontology (GO) terms specific to Biological Process (BP), Cellular Component (CC) and Molecular Functions (MM). A total of 278 genes (log_2_ fold change > 1.5; FDR < 0.05) significantly down-regulated at the 15 h relative to the 5 h time-point were mapped to the GO terms specific for BP, CC and MF pathways. The most enriched GO terms associated with DEGs are depicted in Fig. 2. Out of the 14 GO terms revealed, the five major terms associated with the down-regulated genes were anion transport (GO:0006820), synaptic vesicle localization (GO:0097479), organic anion transport (GO:0015711), secretion (GO:0046903) and signal release (GO:0023061) (Fig. 2a). It should be noted that all of the 14 GO terms were significantly enriched (enrichment pathway *P* value < 0.05) (Fig. 2a). For the functional analysis of genes significantly up-regulated at the 15 h relative to the 5 h time-point, a total of 413 genes were mapped to the GO terms specific for BP, CC and MF pathways. All of the GO terms enriched were significant at an enrichment pathway P value < 0.05 (Fig. 2b). Out of the total 10 GO terms, the five major terms were: regulation of phospholipase activity (GO:0010517), alanine transport (GO:0032328), transmembrane receptor protein tyrosine kinase signaling pathway (GO:0007169), regulation of blood vessel diameter (GO:0097746) and 3′,5′-cyclic-nucleotide phosphodiesterase activity (GO:0004114). Network representation of the enriched GO terms and their associated genes obtained from the mapped genes down-regulated at the 15 h relative to the 5 h time-point is depicted in Fig. 3. Network representation of the enriched GO terms and their associated genes obtained from the mapped genes up-regulated at the 15 h relative to the 5 h time-point is depicted in Fig. 4.

### Hierarchical clustering analysis

**Fig. 5:**
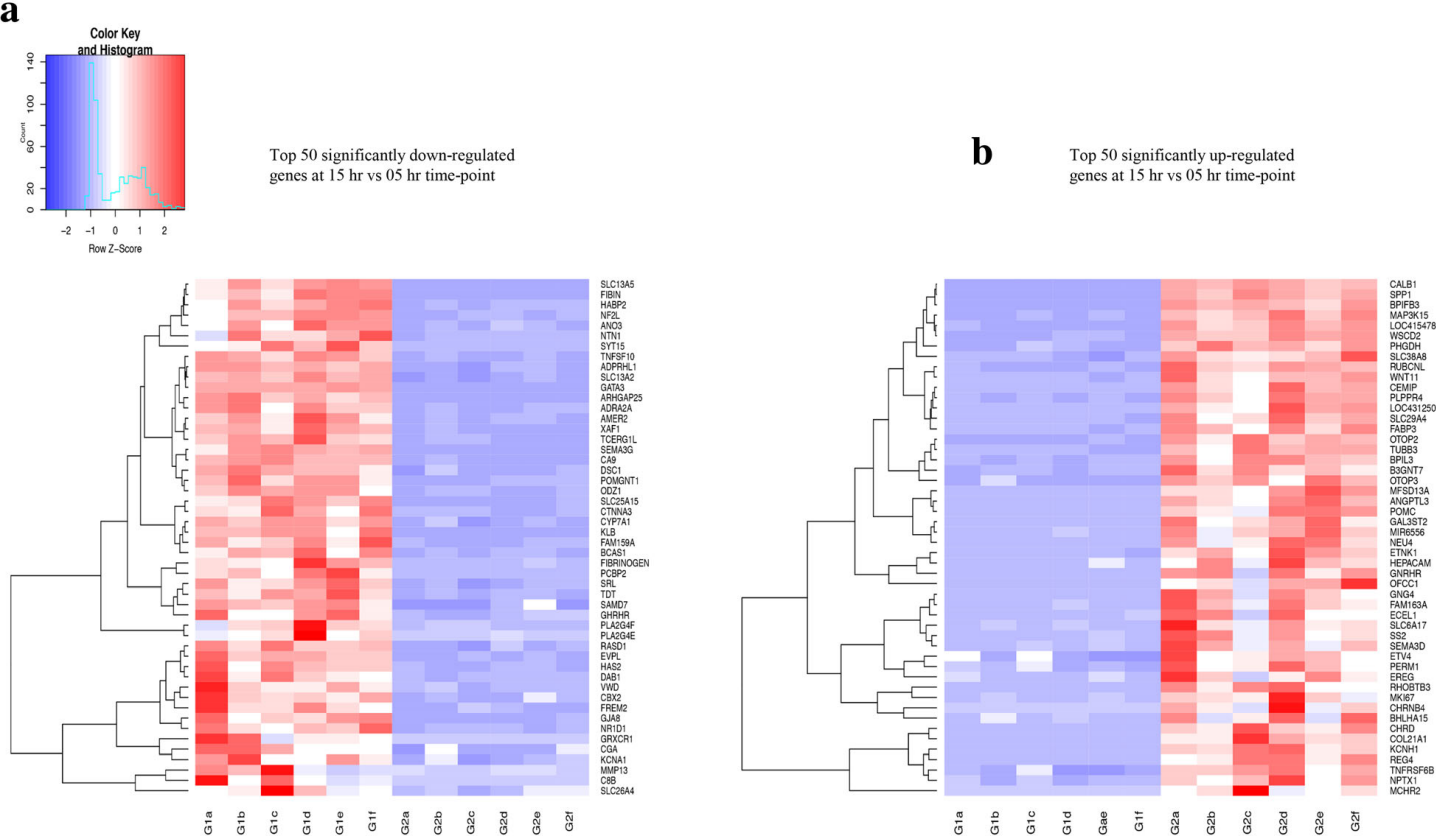
Hierarchical clustering analysis of the top 50 DEGs significantly down- or up-regulated at the 15 h relative to the 5 h time-point. **a** Top 50 DEGs significantly down-regulated at the 15 h relative to the 5 h time-point. **b** Top 50 DEGs significantly up-regulated at the 15 h relative to the 5 h time-point. G1a-f represent six samples taken at the 5 h time-point, while G1a-f represent six samples taken at the 15 h time-point of eggshell formation

Hierarchical clustering analysis (HCA) was performed using the top 50 DEGs down- or up-regulated genes at the 15 h relative to the 5 h time-point of eggshell formation. The pattern of expression for the top 50 DEGs is presented in Fig. 5a, b. A clear difference for the pattern of DEGs at the two time-points has been visualised.

### Validation of RNA-Seq data by qPCR

**Fig. 6:**
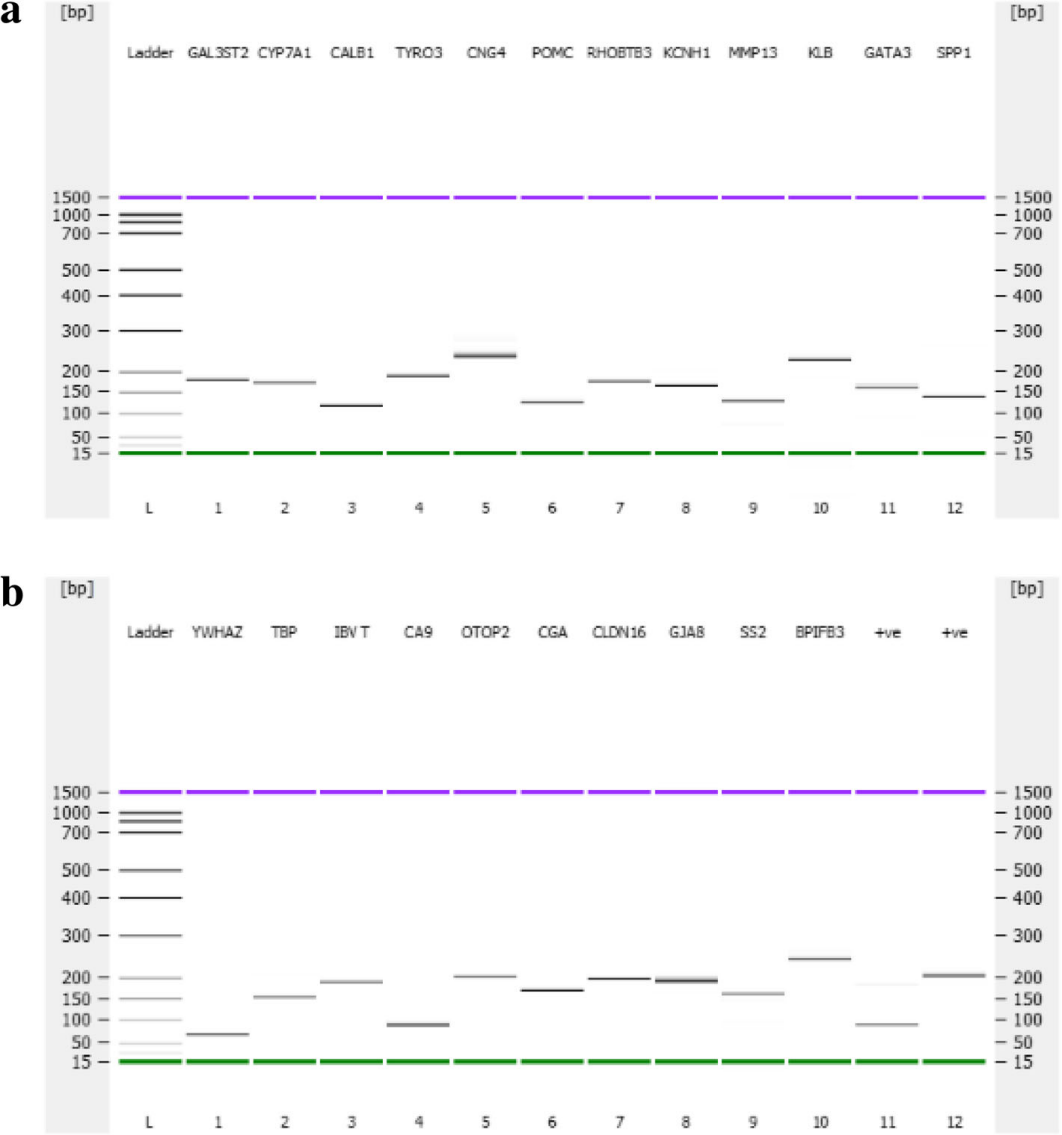
DNA gel electrophoresis of the qPCR products showing that the primers were specific in amplification. Panel **a**: **L**) DNA ladder; **1**) *GAL3ST2* (177 bp); **2**) *CYP7A1* (171 bp); **3**) *CALB1* (116 bp); **4**) *TYRO3* (186 bp); **5**) *CNG4* (243 bp); **6**) *POMC* (115 bp); **7**) *RHOBTB3* (171 bp); **8**) *KCNH1* (160 bp); **9**) *MMP13* (128 bp); **10**) *KLB* (224 bp); **11**) *GATA3* (157 bp); **12**) *SPP1* (134 bp). Panel **b**: **L**) DNA ladder; **1**) *YWHAZ* (61 bp); **2**) *TBP* (147 bp); **3**) IBV T-as positive control (181 bp); **4**) *CA9* (80 bp); **5**) *OTOP2* (202 bp); **6**) *CGA* (166 bp); **7**) *CLDN16* (186 bp); **8**) *GJA8* (186 bp); **9**) *SS2* (160 bp); **10**) *BPIFB3* (234 bp); **11**) *PPARGCIB*-Positive control (82 bp); **12**) *TLR3*-Positive control (203 bp). The upper (purple) and lower markers (green) act as internal standards and are used to align the ladder analysis with the individual DNA sample analysis. The DNA gel in Agilent 2100 Bioanalyzer was performed as per the manufacturer’s instructions of Agilent DNA 1000 Kit. The size of the individual amplicons are consistent with the expected size

Quantitative PCR was performed to validate the significantly down- or up-regulated genes at the 15 h relative to the 5 h time-point obtained in RNA-Seq analysis. All primers used for RNA-Seq data validation by qPCR were specific in amplifications (Fig. 6a, b). The amplification efficiencies of individual primers have been depicted in Table 1. The expression levels of nineteen genes selected for validation of RNA-Seq data showed a positive linear relationship (Table 6). The results suggested that the RNA-Seq is a good reference for expression profiling study and the assembly quality of the sequences was desirable. Although the magnitude of fold change obtained by qPCR and RNA-Seq was slightly different, the qPCR results demonstrated a similar trend (positive correlation) compared with the RNA-Seq for the 19 genes being tested (Table 6).

**Table 6:** Comparison of the gene expression data between RNA-Seq and qPCR

Gene	Fold change	
	qPCR	RNA-Seq
*CGA*	−2.335	−3.078
*CYP7A1*	−10.374	− 4.170
*GATA3*	−7.331	−3.036
*GJA8*	−19.366	−4.522
*MMP13*	−4.320	−4.673
*TYRO3*	−4.249	−1.942
*CA9*	−10.377	−4.519
*KLB*	−38.019	−5.998
*GNG4*	+ 5.427	+ 6.586
*KCNH1*	+ 32.199	+ 5.956
*BPIFB3*	+ 30.038	+ 6.414
*POMC*	+ 316.268	+ 9.179
*SPP1*	+ 306.140	+ 7.993
*GAL3ST2*	+ 68.867	+ 6.999
*OTOP2*	+ 12.366	+ 4.226
*CALB1*	+ 74.817	+ 8.081
*SS2*	+ 13.025	+ 5.434
*CLDN16*	+ 5.378	+ 3.212
*RHOBTB3*	+ 14.644	+ 4.305

For qPCR, the relative expression level of genes at the 5 h and 15 h time-points was calculated in qbase+ software based on 2^^-ΔΔCq^ with genes amplification specific efficiency. For gene expression data normalisation, *TBP* and *YWHAZ* were used as reference genes. Plus and minus signs show down- or up-regulation of genes at the 15 h relative to the 5 h time-point

All the genes tested were either significantly down or up-regulated (*P* < 0.05) at the 15 h relative to the 5 h time-point of eggshell formation. Regression analysis showed a weak positive correlation (R^2^ = 0.526; *P* value 0.004) between the qPCR and RNA-Seq data.

## Discussion

Significant advances have been made in understanding the morphological and biochemical aspects of eggshell biogenesis. However, the molecular mechanisms underpinning the formation of various layers of the eggshell formation are still not well understood. The present study focused on how the regulation of genes was related to eggshell formation by the study of DEGs between the time points when the egg was either in the distal magnum or isthmus (5 h time-point, post oviposition time) or in the shell gland (15 h time-point, post oviposition time). For simplicity of data presentation, the 5 h time-point was taken as reference control to examine the expression changes of the genes at 15 h time-point when the eggshell formation was already initiated. Quantitative PCR results validated RNA-Seq data; therefore, RNA-Seq was used for genome-wide exploration of the gene expression profile of the shell gland. The RNA-Seq analysis revealed many DEGs down- or up-regulated at the 15 h relative to the 5 h time-point of eggshell formation. Some of the genes identified in the current study have been previously implicated in eggshell formation [55]; however, we have also identified multiple new genes that potentially play vital roles during active stages of eggshell formation. In addition, the current study has picturised the expression profile of shell gland when the egg was either in the distal magnum or the distal isthmus, reflecting the preparatory molecular mechanisms occurring in the shell gland. Various layers of eggshell result from the deposition of organic matrix and inorganic minerals secreted to the lumen of shell gland. In the current study, elaborating on molecular mechanisms occurring during eggshell formation; significantly up-regulated genes, such as *CALB1, POMC, SPP1, BPIFB3* and down-regulated genes, such as *SLC13A5, KLB, XAF1* and *MMP13* reflect the differential expression profile of the shell gland during eggshell formation.

DEGs that were significantly up-regulated at the 15 h relative to the 5 h time-point and enriched for GO term pathway analysis showed active stages of eggshell formation. The GO term regulation of phospholipase activity (GO:0010517) shows that the hydrolysis of lipids was higher in order to produce energy for the synthetic processes of eggshell formation. Among the DEGs in phospholipase activity, *CEMIP, ANGPTL3, WNT11, EREG, MAP 3 K15* and *SLC20A1* were significantly up-regulated with log_2_ fold changes of 7.555, 6.198, 4.799, 3.867, 3.736 and 3.302, respectively. *CEMIP* is mainly involved in metabolism, glycosaminoglycan and calcium release metabolism pathways. *CEMIP* interacts with *BIP/HSPA5* for the release of calcium from endoplasmic reticulum [56]. The higher expression levels of *CEMIP* and *HSPA5* (log_2_ fold change 2.403) might indicate their role in calcium release for peak stages of eggshell formation.

*ANGPTL3* is a member of angiopoietin-like (*ANGPTL*) genes that have diverse functions in various pathophysiological [57] and developmental [58] conditions in mammals. The N terminal chain of *ANGPTL3* is also important for lipid metabolism. A higher mRNA expression of *ANGPTL3* was observed in mouse uterus on day 6.5 of pregnancy [59]. In the chicken oviduct, a higher expression of *ANGPTL3* was linked with molecular mechanisms involved in tissue development and remodelling [60]. A significantly higher expression of *ANGPTL3* at the 15 h time-point shows its direct role in eggshell formation. It seems that *ANGPTL3* might have been up-regulated by the release of endocrine hormones involved in molecular mechanisms of eggshell formation and oviposition. *PTN* is among the estrogen stimulating genes, possesses antimicrobial properties [55] and expresses in chicken oviduct [61]. The current study confirms a significant up-regulation of *PTN* during active stages of eggshell formation. The *WNT11* gene functions in developmental processes and its up-regulation at the 15 h time-point (log_2_ fold change 4.799) compared with the 5 h time-point reflects its role in the peak/active stages of eggshell formation. *WNT11* was up-regulated during eggshell formation in laying hens observed in other study [55]. In sheep uterus, *WNT* family encodes signalling regulator molecules vital for cell growth, differentiation and cell-cell interactions [62].

At the 15 h time-point, among the other significantly up-regulated genes were *CALB1, POMC, SPP1, BPIFB3/OCX-36, LOC415478, KCNH1, BPIL3* and *OTOP*3 that have previously been implicated in eggshell formation [55]. A significant up-regulation of *CALB1* at the 15 h (log_2_ fold change 8.081) relative to the 5 h time-point confirms a higher rate of calcium transportation across the cell membrane during the peak stages of eggshell formation. A higher expression of *CALB1* during eggshell calcification in the shell gland and in the intestine of chickens has been reported [4, 33, 55, 63]. Low free Ca^+^ in cells is maintained by calcium uptake in the endoplasmic reticulum through ATP dependent calcium pumps [55]. *ATP2A3* appears to play a role in this Ca^+^ balance, which is confirmed by its up-regulation (log_2_ fold change 3.484) at the 15 h relative to the 5 h time-point. *SPP1* is another important gene involved in eggshell calcification [55, 64]. The peak stages of eggshell formation can be further linked with the significant higher expression of *SPP1* (log_2_ fold change 7.993). A significantly higher expression of *SPP1* was observed between 3 and 20 h post-oviposition times in the shell gland of laying hens by Jeong et al [33]. *SPP1* is involved in bone mineralisation and is present in chicken eggshell [65, 66]. The expression of *SPP1* in chicken uterine tissue is stimulated by the mechanical presence of the forming egg [33, 67]. The gene *POMC* functions in many biological pathways including the stimulation of the release of cortisol hormone. A significant up-regulation (log_2_ fold change 9.179) of the *POMC* at the 15 h time-point highlights its role in the release of hormones necessary for formation of eggshell. A higher expression of *POMC* was observed when a hard shell egg was forming in hen uterine tissue [55].

*BPIFB3* (*OCX-36*) is a lipopolysaccharide-binding protein/bactericidal-permeability increasing protein (LBP/BPI) that is present in various layers of the eggshell and possesses antibacterial activity [25, 55, 68]. In the oviduct of laying chickens, *OCX-36* only expresses in the shell gland [25]. In the current study, a significantly higher expression of *OCX-36* (log_2_ fold change 6.413) at the 15 h vs the 5 h time-point indicates the importance of OCX-36 protein in the shell matrix. A higher expression level of *OCX-36* mRNA has been shown in chicken shell gland in the presence of an egg [25, 55].

The second most enriched GO term at the 15 h time-point, alanine transport (GO:0032328), indicates that the alanine and 2-aminopropanoic acid transport across the shell gland cells was higher, which might be involved in energy (ATP) production during eggshell formation. Significantly up-regulated *SLC6A17* (log_2_ fold change 6.642) at the 15 h relative to the 5 h time-point indicates the importance of the alanine transport pathway during eggshell formation. The transmembrane receptor protein tyrosine kinase signaling pathway (GO:0007169) is usually initiated by the binding of an extracellular ligand to a receptor on the surface of the target cells where the receptor possesses tyrosine kinase activity to regulate transcription. The most enriched GO:0007169 indicates higher transcriptional activities of the cells involved in the synthesis and secretion of macromolecules needed for eggshell formation. The GO enriched term regulation of blood vessel diameter (GO:0097746) suggests that the blood flow to the shell gland at the 15 h time-point was significantly affected by the eggshell formation as has been shown previously [69]. The genes involved in GO term cyclic 3′,5′-phosphodiesterase activity (GO:0004114) encode enzymes that degrade the phosphodiester bond in cAMP and cGMP molecules. The up-regulation of these genes in the shell gland at the 15 h relative to the 5 h time-point indicate their role in energy production during the synthetic activities of shell gland for eggshell formation.

Genes that were significantly down-regulated at the 15 h relative to the 5 h time-point reflect the activities of the shell gland when the egg was forming either in distal magnum or isthmus and was ready to enter to shell gland in the next hour or so. The down-regulated genes annotated to the most enriched GO term anion transport (GO:0006820) indicate that the genes involved in transport of ions across cell membrane were significantly down-regulated in the shell gland. This indicates that the synthesis and secretory activities in the shell gland cells were already initiated, while the egg was still forming in the distal magnum or isthmus. *SLC13A5* (also known as Na^+^/citrate cotransporter) plays an important role in transporting ions and/or molecules across cell membranes. A significantly lower log_2_ fold change (− 6.516) of *SLC13A5* at the 15 h relative to the 5 h time-point might reflect its role in transportation of ions for the initiation of synthesis of molecules necessary for the initiation of eggshell formation. The genes *SLC13A5* and *SLC13A2* belong to solute carrier family 13 group of proteins and are sodium-dependent citrate cotransporters in regulating metabolic processes. Among its related pathways are transport of various sugars, bile salts and organic acids, metal ions and amine compounds. In mammalian cells, *SLC13A5* mediates Na^+^-coupled transport of citrate and succinate for tricarboxylic acid cycle [70]. In the GO term synaptic vesicle localisation, most of the genes involved function in transportation of synaptic vesicles across cell membrane. It seems that the genes in this pathway mainly perform activities in neurotransmission necessary for the transport and synthesis of various molecules including hormones in the shell gland as shown in the present study. Some of the genes that were annotated to the third most enriched GO term, organic anion transport, also served as transporters for organic anions across cell membrane. Organic anions contain molecules that are negatively charged and contain carbon in covalent linkage. The significantly enriched GO term secretion indicates the synthesis of substances that were either directly involved in eggshell formation or served a role in transportation of other molecules such as hormones. The enriched GO term signal release indicates that signal secretion to the extracellular medium from a cellular source was occurring around the 5 h time-point. This may indicate that the shell gland cells were actively involved in the synthesis of molecules necessary for either cellular function or initiation of eggshell formation.

The alignment of the sequences with unknown gene/protein functions suggests that these genes are vital to shell gland function in laying chickens. The majority of the significantly up-regulated DEGs with unknown functions were from the 15 h time-point. It seems that these DEGs were involved in the molecular mechanisms necessary for eggshell formation. We suggest further investigation of their roles in the shell gland relative to egg formation. The associated GO terms with the unknown function genes ranged from calcium ion binding to receptor activity. A large number of novel lincRNA in the current study might indicate their role as regulators in the shell gland of laying hens. Further studies should be performed to investigate the spatio-temporal expression of genes involved in the synthesis of various eggshell layers and the role of microRNA and lincRNA in the regulation of genes involved in eggshell formation.

## Conclusions

Transcriptome analysis revealed thousands of DEGs in shell gland of laying chickens at the 15 h relative to the 5 h time-point of eggshell formation. The significantly down-regulated DEGs indicate that the synthesis activities were already initiated in the shell gland when the egg was still forming in the distal magnum or isthmus regions of the oviduct. The DEGs significantly up-regulated at the 15 h relative to the 5 h time-point reflect the phospholipid activities and synthesis or transport of molecules for the peak period of eggshell formation. The findings in the current study improve our understanding of eggshell formation at molecular level.

## Additional files

Additional file 1: **Table S1.** Egg quality variables measured for dividing experimental hens into two different groups. (DOCX 12 kb)

Additional file 2: **Figure S1.** Multi-dimensional scaling (MDS) plot showing the expression level of genes in 12 different samples. (PDF 27 kb)

## Abbreviations

ACP1: Acid phosphatase 1
CALB1: Calbindin
CYP26A1: Cytochrome P450 family 26 subfamily A member 1
DEGs: Differentially expressed genes
GO: Gene ontology
PENK: Proenkephalin
RCAN1: Regulator of calcineurin 1
RIN: RNA integrity number
SPP1: Secreted phosphoprotein 1
TBP: TATA-Box binding protein
YWHAZ: Tyrosine 3-monooxygenase/Tryptophan 5-monooxygenase activation protein zeta
