# Supplementary material for: RNA-sequencing analysis of shell gland shows differences in gene expression profile at two time-points of eggshell formation in laying chickens
Source: BMC Genomics. 2019 Jan 25;20:89. doi: 10.1186/s12864-019-5460-4 (PMC6347800; doi:10.1186/s12864-019-5460-4)
Supplement: Supplementary file 1 — Table S1. Egg quality variables measured for dividing experimental hens into two different groups. (DOCX 12 kb) [file 12864_2019_5460_MOESM1_ESM.docx]

Table S1: Egg quality variables measured for dividing experimental hens into two different groups.

| **Variable** | **Time-point (hr)** | | **P value** |
| --- | --- | --- | --- |
|  | **05** | **15** |  |
| Shell reflectivity (%) | 16.0±0.37 | 16.1±0.33 | 0.9228 |
| Shell colour (L*) | 54.0±0.42 | 53.9±0.44 | 0.9079 |
| Egg weight (g) | 61.4±1.12 | 62.2±0.88 | 0.5271 |

Eggs analysed for egg quality were collected prior euthanisation of birds. Hens were divided into two different groups (05 hr and 15 hr time-points) in such a way that the two groups were not significantly different for the variables mentioned in Table S1. Values are mean±S.E.
