# Supplementary figures and images for: RNA-sequencing analysis of shell gland shows differences in gene expression profile at two time-points of eggshell formation in laying chickens
Source: BMC Genomics. 2019 Jan 25;20:89. doi: 10.1186/s12864-019-5460-4 (PMC6347800; doi:10.1186/s12864-019-5460-4)

MDS plot (raw)

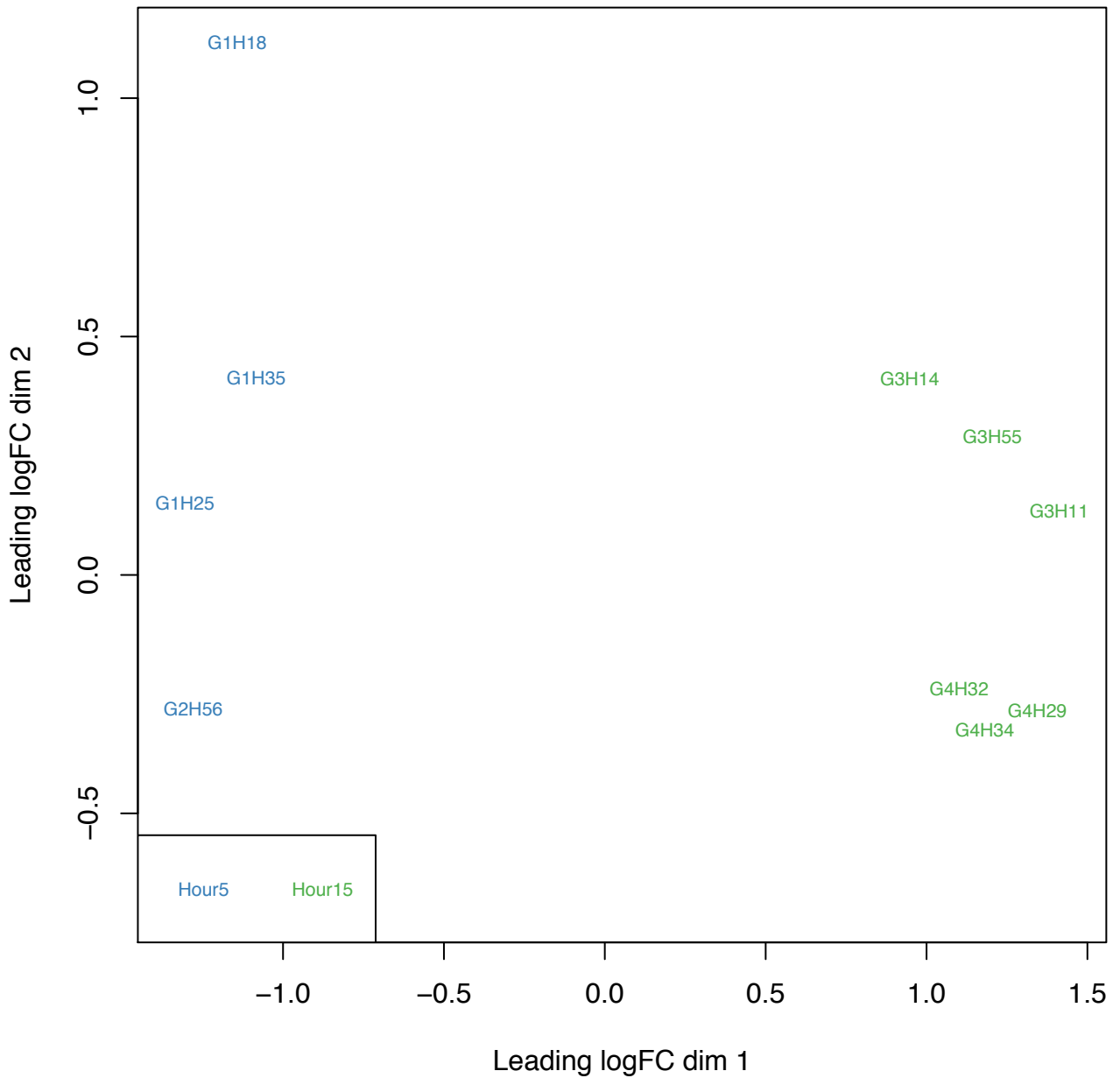

MDS plot (norm)

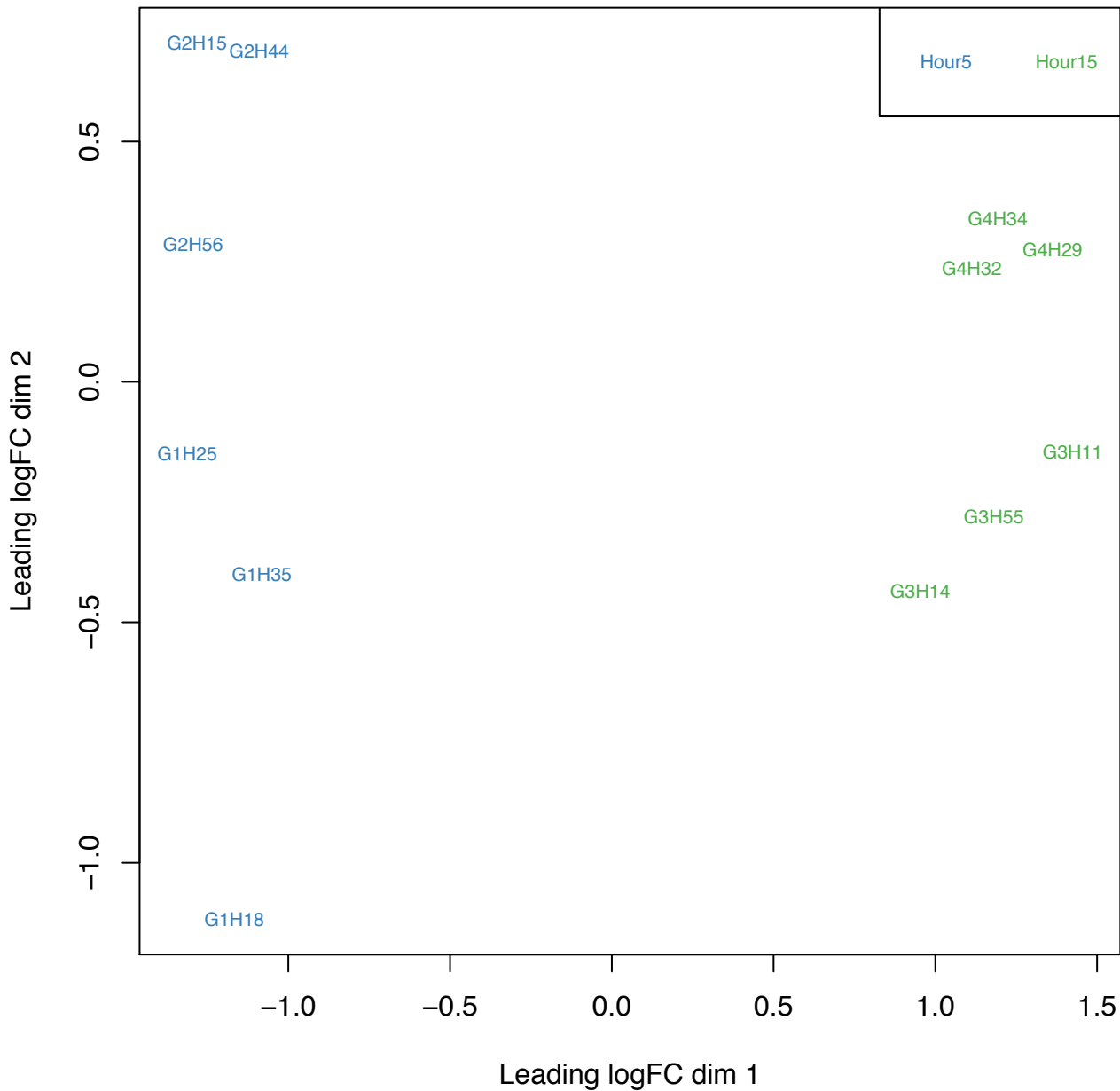

Supplement: Supplementary file 2 — Figure S1. Multi-dimensional scaling (MDS) plot showing the expression level of genes in 12 different samples. (PDF 27 kb) [file 12864_2019_5460_MOESM2_ESM.pdf]
